# Intravenous fosfomycin-containing regimens are associated with reduced acute kidney injury in carbapenem-resistant Gram-negative infections: a retrospective cohort study

**DOI:** 10.3389/fphar.2026.1811298

**Published:** 2026-07-07

**Authors:** Alessandro D’Avino, Elena Paciacconi, Stefania Mazzocchetti, Paolo Dionisi, Daniela Grande, Gabriella Nasi, Marie Claire Theriault, Arcangelo Schiattarella

**Affiliations:** 1 Department of Medical Sciences, IDI IRCCS Istituto Dermopatico dell’Immacolata, Rome, Italy; 2 Intensive Care Unit Department, Aurelia Hospital, Rome, Italy; 3 Health Direction and Risk Management, Cristo Re General Hospital, GIOMI S.p.a., Rome, Italy; 4 Intensive Care Unit Department, Cristo Re General Hospital, GIOMI S.p.a., Rome, Italy; 5 Microbiology and Clinical Pathology Department, Cristo Re General Hospital, GIOMI S.p.a., Rome, Italy

**Keywords:** acute kidney injury (AKI), carbapenem-resistant Gram-negative bacteria (CR-GNB), colistin, combination antibiotic therapy, intravenous fosfomycin

## Abstract

**Background:**

CR-GN (Carbapenem-resistant Gram-negative) infections pose a significant global public health threat due to their high mortality rates. Furthermore, many treatments carry substantial renal toxicity. This study aims to compare renal injury associated with the most commonly used therapeutic regimens in clinical practice.

**Methods:**

A retrospective study was designed selecting patients with invasive CR-GN infections, successfully treated (evideced by microbiological clearance or clinical resolution) in Intensive Care Unit (ICU) and Medicine Department at “Cristo Re” General Hospital in Rome. Renal injury was defined as either a doubling of serum creatinine or a 50% reduction in glomerular filtration rate (GFR) at the end of treatment. Patients on dialysis, those treated for less than 5 days, and those with a baseline GFR of less than 30 mL/min were excluded. Chi-square tests were employed to compare frequencies, and multivariable logistic regression was performed to identify predictors of kidney injury.

**Results:**

We collected data from 59 patients (28 with *Acinetobacter* baumannii, 7 KPC-producing *Klebsiella pneumoniae*, 24 *Pseudomonas aeruginosa*) with a median age of 74 years (IQR 64.8–80), 33 of whom (55.9%) were treated with IV fosfomycin (in combination 28 patients with colistin, 2 pts with colistin and tigecycline and 3 with Ceftazidime/avibactam (CAZ/AVI), 26 patients with other regimens (21 of which including colistin). The median treatment duration was 10 days (IQR 7–14). At the end of treatment, 11 patients (18.6%) developed acute kidney injury, with a statistically significant difference favoring fosfomycin containing regimens (9.1% vs. 30.8% P = 0.037). Among colistin-containing regimens, the renal injury rate was significantly lower when fosfomycin was included (10.7% vs. 33.3%, P = 0.05). Logistic regression analysis identified the use of fosfomycin as an independent predictor of kidney injury prevention (OR = 0.074; IC 0.008–0.678 p = 0.021).

**Conclusion:**

This study confirms that regimens used for treating carbapenem-resistant Gram-negative bacterial infections cause a high rate of acute kidney injury, largely due to the frequent use of known nephrotoxic drugs, like colistin. However, our data suggest that combining these drugs with intravenous fosfomycin may offer renal protective benefits.

## Introduction

Antimicrobial resistance is profoundly reshaping the management of infectious diseases. In particular, infections caused by carbapenem-resistant Gram-negative bacteria represent a major therapeutic challenge, as the available treatment options are limited and often burdened by significant toxicity, especially in critically ill patients.

In 2017, the WHO prioritized carbapenem-resistant Gram-negative (CR-GN) bacteria as critical pathogens in its ranking of antibiotic-resistant threats (World Health Organization, 2017). The 2022 report of the European Antimicrobial Resistance Surveillance Network (EARS-Net), coordinated by the European Centre for Disease Prevention and Control, provides surveillance data on antimicrobial resistance in invasive bacterial isolates across European Union/European Economic Area (EU/EEA) countries.

According to this report, carbapenem resistance remains rare in *Escherichia coli*, whereas resistance rates above 10% were reported for *Klebsiella pneumoniae* in nearly one-third of EU/EEA countries. Carbapenem resistance also remains a major concern in *Pseudomonas aeruginosa* and *Acinetobacter* spp. (European Centre for Disease Prevention and Control, 2023).

CR-GN infections represent a global public health threat because of their high mortality rates. The impact of these infections on clinical outcomes is well documented. An international study reported that 62% of ICU infections were caused by multidrug-resistant (MDR) Gram-negative bacteria, with mortality rates among MDR-infected patients more than twice those observed in non-infected patients (25% vs. 11%) (Vincent JL et al., 2009). Similar findings were observed in a large cohort study conducted in Greece, where the mortality rate for CR-GN infections reached 49.3% (Kousouli et al., 2019).

The global threat posed by CR-GN infections is further exacerbated by the limited therapeutic options available, many of which are associated with significant renal toxicity. Nephrotoxic antibiotics, such as polymyxins and aminoglycosides, are broadly used as part of combination regimens to treat CR-GN infections. Although new agents with proven efficacy have recently been introduced (Isler et al., 2018; Bassetti et al., 2018), *in vitro* studies suggest a potential role for intravenous fosfomycin, an older antibiotic, in the treatment of CR-GN infections (Ku et al., 2019; Flamm et al., 2019; Singkham-In and Chatsuwan, 2018). Recent clinical studies have shown that fosfomycin-containing regimens may reduce all-cause mortality in patients with MDR pneumonia caused by *Acinetobacter* baumannii (Jung et al., 2017; Russo et al., 2021).

We hypothesized that, beyond its synergistic activity with other antimicrobials, the improved outcomes associated with fosfomycin use in critically ill patients may also be related to its favorable safety profile and lower nephrotoxicity. Therefore, this study aimed to compare the occurrence of renal injury among patients with CR-GN infections treated with different antibiotic regimens, with particular attention to the potential protective role of fosfomycin.

## Methods

We designed a retrospective observational study conducted in Italy in a 300-bed hospital in Rome. We collected data of patients hospitalized from December 2018 to February 2022, all diagnosed with a CR-GN infection. Inclusion criteria included: 1) Age>18 years; 2) Culture positive for a CR-GN strain; 3) clinical signs and symptoms consistent with pneumonia, hospital-acquired pneumonia (HAP) and ventilator-acquired pneumonia (VAP) included, urinary tract infections (UTI), bacteremia (BSIs) or surgical intra-abdominal infections (IAIs) 4) Microbiological clearance or clinical cure of CR-GN infection (defined as resolution of signs and symptoms of infection or survival at 28 days). Patients with polymicrobial etiology, those on dialysis, treated for less than 5 days or with a baseline GFR <30 mL/min were excluded. Only one episode of CR-GN infection per patient was reported.

To focus on the tolerability of regimens, we included only patients in targeted antibiotic therapy, excluding those receiving empiric therapy without bacterial isolation or those whose empirical regimens were found to be inappropriate after culture results. Accordingly, all isolates were susceptible to the antibiotics used. Because of the retrospective design and the absence of a formal screening log, the total number of CR-GNB cases initially assessed during the study period and the number of patients excluded at each selection step could not be reliably reconstructed. The final study population consisted of 59 patients who fulfilled all inclusion criteria and none of the predefined exclusion criteria. Due to the retrospective nature of the study, the Ethics Commitee (Lazio 1) waived the need for informed consent. The study adhered to the principles stated in the Declaration of Helsinki. Patient data were collected from medical charts and from hospital computerized databases or clinical charts according to the protocol. The following information were collected: demographics; clinical features; laboratory findings at baseline and at the end of treatment; comorbid conditions; microbiologic data; duration of hospital stay; history of CR-GN infection, previous hospitalization within 180 days before the index CR-GN infection, site of infection, presence of septic shock at diagnosis (with use of vasopressors included), treatment regimen, duration of therapy and all-cause mortality during hospitalization. Conversely, the exact duration of hospitalization before ICU admission and complete data on empirical antimicrobial therapy before pathogen identification were not systematically available for all patients and were therefore not included in the statistical analysis.

Infections were defined according to the definitions of the European Centre for Disease Control and Prevention (eCDC) (European Centre for Disease Prevention and Control, 2014). Infection was characterized by at least one positive culture from, lung, urine, blood or abdominal drainage for CR-GN in individuals with signs and symptoms consistent with pneumonia, UTI, IAI or sepsis. HAP was considered pneumonia occurring 48 h or more after admission that did not appear to be incubating at the time of admission. VAP was defined as HAP developing 48h after endotracheal intubation. UTIs included infections in any part of the urinary system, including the kidneys, ureters, bladder, and urethra (Hooton, 2012). Surgical site infections were defined as infections that occur at or near a surgical incision site within 30 days after surgery or within 1 year if an implant is left in place (Mangram et al., 1999).

Septic shock was defined as a subset of sepsis meeting the following criteria: 1) suspected or documented infection, an acute increase in Sequential [Sepsis-related] Organ Failure Assessment (SOFA) score of 2 points or more, and persistent hypotension requiring vasopressors to maintain a mean arterial pressure (MAP) of 65 mmHg or greater, alongside a serum lactate level greater than 2 mmol/L despite adequate volume resuscitation (Singer et al., 2016).

Renal injury was assessed using serum creatinine-based criteria for acute kidney injury (AKI), in line with the KDIGO framework. Owing to the retrospective design of the study and the lack of systematic urine output data, AKI was defined as a doubling of serum creatinine from baseline at the end of treatment, corresponding to KDIGO stage 2 AKI. A ≥50% reduction in estimated glomerular filtration rate was also considered, consistent with previous RIFLE-based definitions of renal injury (Bellomo et al., 2004; Mehta et al., 2007; KDIGO Clinical Practice Guideline for Acute Kidney Injury, 2012). Because of the retrospective design of the study, serial renal function measurements during the treatment course were not consistently available for all patients. Therefore, AKI was assessed by comparing renal function at baseline and at the end of antimicrobial treatment.

Local laboratory techniques, specifically the Vitek 2 automated system (bioMérieux, Marcy l’Etoile, France), were used for isolate identification and antimicrobial susceptibility testing, except for colistin and fosfomycin, whose susceptibility was confirmed via the Kirby-Bauer test. Minimum inhibitory concentrations (MICs) were determined according to the European Committee on Antimicrobial Susceptibility Testing (EUCAST) breakpoints (European Committee on Antimicrobial Susceptibility Testing EUCAST, 2020).

Antibiotic regimens were selected according to Infectious diseases (ID) specialist consultation according to cultures collected during the study period for each patient.

Standard dosages were employed for most antibiotics, as follows: colistin, a loading dose of 9 million IU followed by 4.5 million IU every 12 h, or reduced up to 2 million IU every 12 h based on renal clearance; tigecycline, a loading dose of 200 mg followed by 100 mg every 12 h; amikacin, a dosage of 15 mg/kg every 24 h; gentamicin a dosage of 5 mg/kg every 24 h; meropenem, a dosage of 2 g every 8 h or 1 g tid; fosfomycin 12–24 g/day divided every 6–8 h; ceftazidime/avibactam a dosage of 2.5 g every 8 h; trimethoprim/sulfamethoxazole 15–20 mg/kg/day divided every 6 h. Therapy was administered for a minimum of 5 days, and patients who died before 28 days post-treatment were excluded.

### Primary endpoint and statistical analysis

Primary endpoint was to evaluate the rate of renal injury in patients successfully treated for CR-GN infections. Subgroup analyses were performed to compare intravenous fosfomycin-containing regimens with fosfomycin-sparing regimens. We also analyzed the renal injury rate in patients treated with colistin, both alone and in combination with other antimicrobials.

Baseline characteristics of patients were described as frequencies for categorical variables and as median and IQR for continuous variables. To detect significant differences between groups we used chi-square test for categorical variables, the U-Mann Whitney test to compare means, and multivariable logistic regression to evaluate independent predictors of kidney injury. Statistical significance was set at P ≤ 0.05. All reported P values were two-tailed. The results obtained were analyzed using a commercially available statistical software package (SPSS, version 23.0; SPSS Inc., Chicago, IL).

## Results

During the study period we collected data from 59 patients admitted at “Cristo Re -General Hospital, GIOMI S. p.A in Rome” in ICU department and Internal Medicine ward. Baseline characteristics were shown in Table 1. Most patients (62.7%) were admitted in ICU department. All the patients were treated for invasive infection due to multidrug resistant Gram-negative bacteria, 28 patients (47.5%) with *Acinetobacter* baumannii, 7 patients (11.9%) with KPC-producing *Klebsiella pneumoniae* and 24 patients (40.7%) with MDR *Pseudomonas aeruginosa*. At the baseline 14/59 patients (23.7%) showed a reduction of renal clearance within the limit of 30 mL/min according to the Study protocol. Most patients were treated for pneumonia (hospital-acquired pneumonia 11 pts, ventilator-acquired pneumonia 22 pts), followed by blood stream infections (17/59 pts) and urinary tract infections (15/59 pts).

**TABLE 1:** Baseline characteristics.

Patients	59
Age (median years, IQR)	74 (64.9–80)
Sex male (n,%)	37 (62.7)
MDRO (n, %) Acinetobacter baumannii KPC Pseudomonas aeruginosa	28 (47.5) 7 (11.9) 24 (40.7)
Comorbidities (n, %) Cardiopathy any Diabetes Renal impairment at baseline (clearance from 39 to 50 mL/min/1.73 mq) COPD Cancer Alcohol abuse Liver impairment	28 (47.5) 22 (37.3) 14 (23.7) 31 (52.5) 10 (16.9) 3 (5.1) 2 (3.4)
Infection (n, %) HAP VAP UTI BSI Surgical intra abdominal infection	11 (18.6) 22 (37.3) 15 (25.4) 17 (28.8) 3 (5.1)
Median duration of therapy (median days, IQR)	10 (7–10)
Septic shock at baseline (n, %)	13 (22)
Previous MDR related infections (n, %)	23 (39)
History of hospitalization 180 days before (n, %)	41 (69.5)

Abbreviations: BSI, bloodstream infection; COPD, chronic obstructive pulmonary disease; HAP, hospital-acquired pneumonia; IQR, interquartile range; KPC, *Klebsiella pneumoniae* carbapenemase; MDRO, multidrug-resistant organism; UTI, urinary tract infection; VAP, ventilator-associated pneumonia.

Table 2 presents a univariate analysis comparing patients treated with fosfomycin containing-regimens and those who received other combinations. Significant differences were noted in ICU admission (75.8% vs. 46.2% in favor of fosfomycin group, P = 0.02). Patients treated with fosfomycin, also, were more likely to have a history of COPD (66.7% vs. 34.6%, P = 0.014), hypertension (75.8% vs. 50%, P = 0.04), a diagnosis of septic shock at baseline (33.3% vs. 7.7%, P = 0.018). Groups were homogeneous for renal impairment at the baseline.

**TABLE 2:** Differences among groups.

Variable (n,%)	Fosfomycin sparing group (26 patients)	Fosfomycin containing group (33 patients)	*P* value
Sex male	16 (61.5)	21 (63.6)	0.865
ICU admission	**12 (46.2)**	**25 (75.8)**	**0.020**
Cardiopathy	9 (34.6)	19 (57.6)	0.08
Diabetes	5 (19.2)	9 (27.3)	0.471
Renal impairment	9 (34.6)	13 (39.4)	0.706
Liver impairment	0 (0)	2 (6.1)	0.202
Alcohol abuse	2 (7.7)	1 (3)	0.418
Cancer	6 (23.1)	4 (12.1)	0.265
COPD	**9 (34.6)**	**22 (66.7)**	**0.014**
Hypertension	**13 (50)**	**25 (75.8)**	**0.040**
HAP	5 (19.2)	6 (18.2)	0.918
BSI	8 (30.8)	9 (27.3)	0.708
UTI	**10 (38.5)**	**5 (15.2)**	**0.041**
VAP	**6 (23.1)**	**16 (48.5)**	**0.045**
Septic shock	**2 (7.7)**	**11 (33.3)**	**0.018**
Age (median, IQR)	73 (60.7–82)	74 (68.6–80)	0.731

Abbreviations: BSI, bloodstream infection; COPD, chronic obstructive pulmonary disease; HAP, hospital-acquired pneumonia; ICU, intensive care unit; IQR, interquartile range; UTI, urinary tract infection; VAP, ventilator-associated pneumonia.

Bold values indicate statistically significant p‐values (p < 0.05).

Treatment regimens were heterogeneous and distributed as follows: the most commonly used regimen was colistin plus fosfomycin (28 patients, 48%), 8 patients (13.5%) were treated with Colistin plus carbapenems (imipenem or meropenem), 6 (10.1%) with colistin in monotherapy. Other regimens included: CAZ/AVI plus colistin (2 pts); CAZ-AVI plus fosfomycin (2 pts); CAZ-AVI monotherapy (2 pts); CAZ/AVI plus meropenem (2 pts) CAZ/AVI plus colistin plus fosfomycin (1 pt); Colistin plus tigecycline (2 pts); colistin plus fosfomycin plus tigecycline (1 pt); fosfomycin plus tigecycline (1 pt); fosfomycin plus amikacin (1 pt); colistin plus gentamicin (1 pt), trimethoprim/sulfamethoxazole (1 pt); colistin plus tigecycline plus meropenem (1 pt). In our sample, fosfomycin was primarily used to treat ventilator-associated pneumonia (48.5% vs. 23.1%, P = 0.045), while fosfomycin-sparing combinations were preferred for urinary tract infections (15.2% vs. 38.5%, P = 0.041).

**FIGURE 1:**
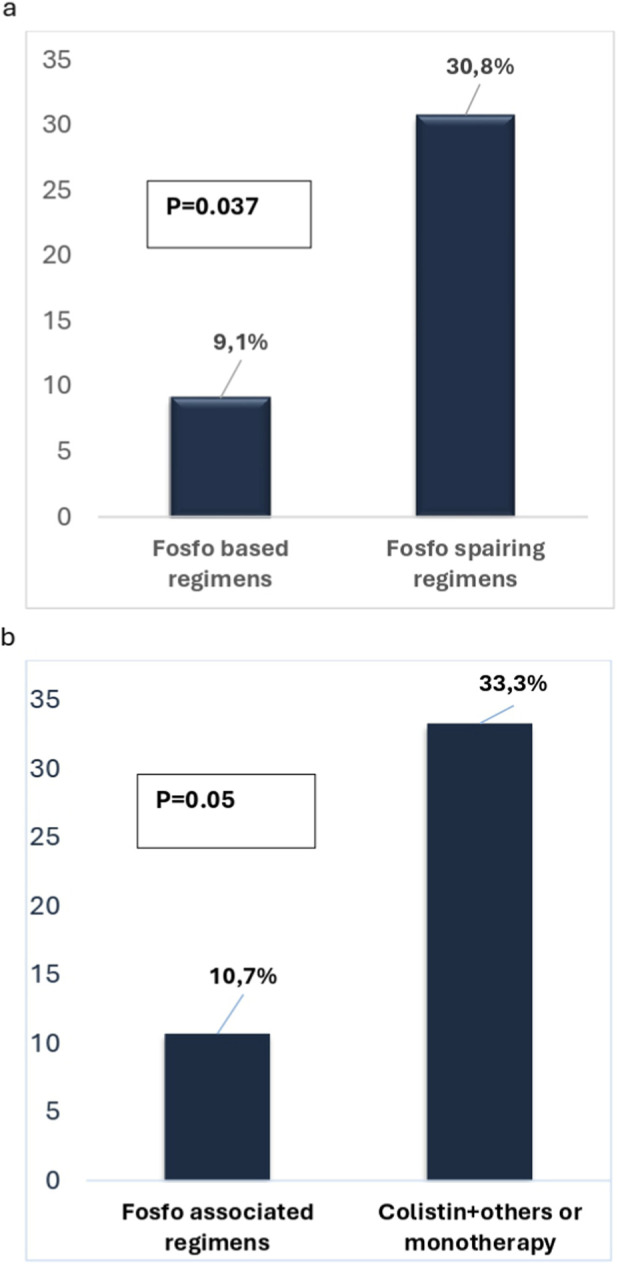
Outcomes. **(a)** Renal failure caused by therapy among groups. **(b)** Subgroup of patients treated with colistin.

The median duration of treatment was 10 days (IQR 7–14). At the end of treatment, 11 patients (18.6%) developed acute kidney injury, with a statistically significant difference in favor of fosfomycin containing regimens (9.1% vs. 30.8%, P = 0.037). When analyzing only colistin-containing regimens, the rate of renal injury was significantly lower when fosfomycin was included (10.7% vs. 33.3%, P = 0.05) (Figure 1). Among patients receiving colistin-containing regimens, the duration of colistin therapy did not differ significantly between patients treated with colistin plus fosfomycin and those receiving colistin-containing regimens without fosfomycin. The mean duration of colistin exposure was 11.04 days in the colistin plus fosfomycin group and 11.48 days in the colistin without fosfomycin group (mean difference −0.44 days; 95% CI −2.47 to 1.59; p = 0.665). According to the protocol, all patients completed the treatment with clinical or microbiological success. However, as a secondary endpoint, we also evaluated all-cause mortality during hospitalization, which was 13.8% (8 patients), without any statistically significant differences among the regimens used.

In a multivariable logistic regression (Table 3), given the limited number of AKI events, a parsimonious model including only septic shock and fosfomycin exposure was performed. In this model, septic shock was independently associated with an increased risk of AKI (OR = 10.368; 95% CI 1.070–100.490; p = 0.044), whereas fosfomycin exposure remained independently associated with a lower risk of AKI (OR = 0.074; 95% CI 0.008–0.678; p = 0.021). This finding should not be interpreted as being primarily related to vasopressor therapy itself, since vasopressors such as noradrenaline are essential to maintain adequate mean arterial pressure and preserve organ perfusion in patients with shock. Rather, septic shock likely reflects a complex and multifactorial risk condition for AKI, including hemodynamic instability, systemic inflammation, microcirculatory dysfunction, impaired renal perfusion, and concomitant exposure to nephrotoxic antimicrobials.

**TABLE 3:** Multivariable logistic regression for renal failure.

Variable	OR (exp (B))	95% CI	p-value
Septic shock	10.368	1.070–100.490	0.044
Fosfomycin exposure	0.074	0.008–0.678	0.021

## Discussion

In this study we evaluated the effect of intravenous fosfomycin on renal function, in critically ill patients with infections due to multidrug resistant Gram negative microorganisms. Our data confirmed a lower rate of renal injury in patients treated with fosfomycin containing regimens.

The potential protective effect of fosfomycin on renal clearance has been demonstrated in previous studies.

One evaluated the effect of fosfomycin and imipenem-cilastatin on nephrotoxicity of vancomycin and cisplatin in rats (Nakamura T et al., 1999). In this model, when fosfomycin was used, a decrease in accumulation of vancomycin was detected in rats. Additionally, co-administration of fosfomycin and cisplatin injection, was associated with lower plasma levels of N-acetyl-β-D-glucosaminidase, a known marker of renal impairment.

Another study suggested a different nephroprotective mechanism of fosfomycin when administered with aminoglycosides in rat renal tissue. In this murine model fosfomycin reduced lipid peroxidation caused by gentamicin, by inhibiting iron release from mitochondria (Yanagida C et al., 2004).

Furthermore in rats, Fosfomycin decreased glycopeptide antibiotic-induced nephrotoxicity evidenced by reduced urinary excretion of N-acetyl-beta-D-glucosaminidase (NAG) in groups treated with a combination of vancomycin or teicoplanin and fosfomycin compared to glycopeptide alone. Notably, higher doses of fosfomycin were correlated with greater reductions in urinary NAG levels, suggesting a dose-dependent effect (Yoshiyama Y et al., 2001).

Given these findings, oral fosfomycin has often been used as an ancillary therapy in oncologic patients treated with cisplatin.

The synergistic effect of fosfomycin with other antimicrobials is already well-documented. A recent study, conducted on KPC strains, showed a synergy rate of 66.7% in ceftazidime-avibactam resistant and fosfomycin-sensitive isolates, while the rate was 42.3% in ceftazidime-avibactam-sensitive fosfomycin-resistant isolates (Tüzemen et al., 2024).

However the hypothesis of synergistic effect of fosfomycin and ceftazidime-avibactam remains controversial with some studies reporting no synergy (Romanelli et al., 2020) and others indicating a synergistic effect (Mikhail et al., 2019).

In addition “*in vivo*” studies failed to show a better outcome, managing infections caused by KPC strains for critically ill patients, treated with combination regimens of ceftazidime-avibactam plus meropenem and fosfomycin as compared to monotherapy, in terms of 14 and 30-day mortality (Önal et al., 2024).

In contrast, more consistent evidence supports a synergistic effect of fosfomycin combined with colistin, particularly against *Acinetobacter* baumannii strains (Ontong JC et al., 2021; Wang J et al., 2018; Nwabor OF et al., 2021).

Indeed, combination therapy is generally preferred for *Acinetobacter* baumannii infections, and fosfomycin-containing regimens represent a viable option.

Our study aimed to determine whether the improved outcomes could be solely attributed to the previously described synergistic effects, or if fosfomycin also offers a better safety profile when combined with nephrotoxic drugs, as demonstrated *in vitro* and in animal studies. To our knowledge, this is the first study analyzing the effect of fosfomycin on renal function when combined with other antimicrobials “*in vivo*” in a real-world setting.

The analysis has limitations. Firstly, the retrospective observational design of the study, and small sample size may have introduced biases. Second, although we had access to medical history for all patients, we lacked data on concomitant oral drug exposure, which could have interfered with the assessment of renal function in both groups. Another limitation is that the retrospective design did not allow a systematic evaluation of the temporal course of renal function during antimicrobial therapy. In particular, serial serum creatinine and estimated glomerular filtration rate values were not uniformly available for all patients; therefore, renal injury was assessed only by comparing baseline and end-of-treatment values. This limits our ability to define the exact timing, progression, or reversibility of AKI during treatment. Lastly, study groups were not perfectly homogeneous for baseline characteristics as a “propensity score matching” was not performed. Nonetheless, despite differences in baseline characteristics, including a higher proportion of patients with COPD, hypertension, and septic shock in the fosfomycin-containing group, the rate of AKI was lower in patients receiving fosfomycin. Septic shock should be interpreted as a major multifactorial risk condition for AKI, reflecting hemodynamic instability, systemic inflammation, impaired renal perfusion, and exposure to nephrotoxic agents. Therefore, although our findings suggest a potential renal safety advantage of fosfomycin-containing regimens, this association should be interpreted with caution because residual confounding cannot be excluded.

## Conclusion

In conclusion, our real-life experience regarding the effect of intravenous fosfomycin on renal function provides valuable insights for clinicians managing infections caused by carbapenem-resistant Gram-negative microorganisms. Renal impairment in these patients poses a crucial challenge especially when they are concurrently exposed to other nephrotoxic drugs, particularly those who develop septic shock. In this context fosfomycin could serve as a valuable companion to other antimicrobials in treating these difficult-to-manage infections.

Our data suggest a potential protective effect of fosfomycin on renal clearance, consistent with findings from several *in vitro* and murine model studies. However, this observation should be interpreted with caution, as the occurrence of AKI in critically ill patients with CR-GN infections is strongly influenced by septic shock and other non-antibiotic-related factors. Therefore, the association between fosfomycin-containing regimens and reduced AKI should be confirmed in further randomized clinical trials.

## Data Availability

The raw data supporting the conclusions of this article will be made available by the authors, without undue reservation.

## References

[B1] Bassetti M. Righi E. Russo A. Carnelutti A. (2018). New antibiotics for pneumonia. Clin. Chest Med. 39, 853–869. 10.1016/j.ccm.2018.08.007 30390754

[B2] Bellomo R. Ronco C. Kellum J. A. Mehta R. L. Palevsky P. Acute Dialysis Quality Initiative workgroup (2004). Acute dialysis quality initiative workgroup acute renal failure - definition, outcome measures, animal models, fluid therapy and information technology needs: the second international consensus conference of the acute dialysis quality initiative (ADQI) group. Crit. Care 8, R204–R212. 10.1186/cc2872 15312219 PMC522841

[B3] European Centre for Disease Prevention and Control (2014). Annual Epidemiological Report on Communicable Diseases in Europe. Stockholm, Sweden: European Centre for Disease Prevention and Control. Available online at: https://ecdc.europa.eu/sites/portal/files/media/en/publications/Publications/AER-VPD-IBD2014.pdf (Accessed July 6, 2020). 22114980

[B4] European Centre for Disease Prevention and Control (2023). Antimicrobial Resistance in the EU/EEA (EARS-Net) - Annual Epidemiological Report 2022. Stockholm: ECDC. Available online at: https://www.ecdc.europa.eu/en/publications-data/surveillance-antimicrobial-resistance-europe-2022 (Accessed February 10, 2026).

[B5] European Committee on Antimicrobial Susceptibility Testing (EUCAST) (2020). Available online at: https://www.eucast.org/clinical_breakpoints (Accessed July 6, 2020).

[B6] Flamm R. K. Rhomberg P. R. Lindley J. M. Sweeney K. Ellis-Grosse E. J. Shortridge D. (2019). Evaluation of the bactericidal activity of fosfomycin in combination with selected antimicrobial comparison agents tested against gram-negative bacterial strains by using time-kill curves. Antimicrob. Agents Chemother. 63, e02549–e02618. 10.1128/AAC.02549-18 30858207 PMC6496043

[B7] Hooton T. M. (2012). Uncomplicated urinary tract infection. N. Engl. J. Med. 366 (11), 1028–1037. 10.1056/NEJMcp1104429 22417256

[B8] Isler B. Doi Y. Bonomo R. A. Paterson D. L. (2018). New treatment options against carbapenem-resistant Acinetobacter baumannii infections. Antimicrob. Agents Chemother. 63, e01110. 10.1128/AAC.01110-18 30323035 PMC6325237

[B9] Jung S. Y. Lee S. H. Lee S. Y. Yang S. Noh H. Chung E. K. (2017). Antimicrobials for the treatment of drug-resistant Acinetobacter baumannii pneumonia in critically Ill patients: a systemic review and bayesian network meta-analysis. Crit. Care 21, 319. 10.1186/s13054-017-1916-6 29262831 PMC5738897

[B10] Kousouli E. Zarkotou O. Polimeri K. Themeli-Digalaki K. Pournaras S. (2019). Impact of bloodstream infections caused by carbapenem-resistant Gram-negative pathogens on ICU costs, mortality and length of stay. Infect. Prev. Pract. 1 (2), 100020. 10.1016/j.infpip.2019.100020 34368681 PMC8335918

[B11] Ku N. S. Lee S. H. Lim Y. S. Choi H. Ahn J. Y. Jeong S. J. (2019). *In* vivo efficacy of combination of colistin with fosfomycin or minocycline in a mouse model of multidrug-resistant Acinetobacter baumannii pneumonia. Sci. Rep. 9, 17127. 10.1038/s41598-019-53714-0 31748527 PMC6868184

[B12] Mangram A. J. Horan T. C. Pearson M. L. Silver L. C. Jarvis W. R. The Hospital Infection Control Practices Advisory Committee (1999). Guideline for prevention of surgical site infection, 1999. Am. J. Infect. Control 27 (2), 97–132. 10.1016/S0196-6553(99)70088-X 10196487

[B13] Mehta R. L. Kellum J. A. Shah S. V. Molitoris B. A. Ronco C. Warnock D. G. (2007). Acute kidney injury network acute kidney injury network: report of an initiative to improve outcomes in acute kidney injury. Crit. Care 11, R31. 10.1186/cc5713 17331245 PMC2206446

[B14] Mikhail S. Singh N. B. Kebriaei R. Rice S. A. Stamper K. C. Castanheira M. (2019). Evaluation of the synergy of ceftazidime–avibactam in combination with meropenem, amikacin, aztreonam, colistin, or fosfomycin against well-characterized multidrug-resistant Klebsiella pneumoniae and Pseudomonas aeruginosa. Antimicrob. Agents Chemother. 63 (8), e00779–e00819. 10.1128/AAC.00779-19 31182535 PMC6658738

[B15] Nakamura T. Kokuryo T. Hashimoto Y. Inui K. I. (1999). Effects of fosfomycin and imipenem-cilastatin on the nephrotoxicity of vancomycin and cisplatin in rats. J. Pharm. Pharmacol. 51 (2), 227–232. 10.1211/0022357991772187 10217324

[B16] Nwabor O. F. Terbtothakun P. Voravuthikunchai S. P. Chusri S. (2021). Evaluation of the synergistic antibacterial effects of fosfomycin in combination with selected antibiotics against carbapenem-resistant Acinetobacter baumannii. Pharm. (Basel) 14 (3), 185. 10.3390/ph14030185 33668905 PMC7996625

[B17] Önal U. Tüzemen Ü. Küçükdemirci Kaya P. İşçimen R. Kelebek Girgin N. Özakın C. (2024). A comparative study of ceftazidime/avibactam-based and fosfomycin plus meropenem-based regimens for managing infections caused by carbapenem-resistant Klebsiella pneumoniae in critically ill patients. J. Chemother. 37, 1–9. 10.1080/1120009X.2024.2349439 38698711

[B18] Ontong J. C. Ozioma N. F. Voravuthikunchai S. P. Chusri S. (2021). Synergistic antibacterial effects of colistin in combination with aminoglycoside, carbapenems, cephalosporins, fluoroquinolones, tetracyclines, fosfomycin, and piperacillin on multidrug resistant Klebsiella pneumoniae isolates. PLoS One 16 (1), e0244673. 10.1371/journal.pone.0244673 33406110 PMC7787437

[B19] Romanelli F. De Robertis A. Carone G. Dalfino L. Stufano M. Del Prete R. (2020). In vitro activity of ceftazidime/avibactam alone and in combination with fosfomycin and carbapenems against KPC-producing Klebsiella pneumoniae. New Microbiol. 43 (3), 136–138. 32596740

[B20] Russo A. Bassetti M. Bellelli V. Bianchi L. Marincola Cattaneo F. Mazzocchetti S. (2021). Efficacy of a Fosfomycin-Containing regimen for treatment of severe pneumonia caused by multidrug-resistant acinetobacter baumannii: a prospective, observational study. Infect. Dis. Ther. 10 (1), 187–200. 10.1007/s40121-020-00357-8 33068255 PMC7568458

[B21] Singer M. Deutschman C. S. Seymour C. W. Shankar-Hari M. Annane D. Bauer M. (2016). The third international consensus definitions for sepsis and septic shock (Sepsis-3). JAMA 315 (8), 801–810. 10.1001/jama.2016.0287 26903338 PMC4968574

[B22] Singkham-In U. Chatsuwan T. (2018). *In* vitro activities of carbapenems in combination with amikacin, colistin, or fosfomycin against carbapenem-resistant Acinetobacter baumannii clinical isolates. Diagn Microbiol. Infect. Dis. 91, 169–174. 10.1016/j.diagmicrobio.2018.01.008 29433997

[B23] Tüzemen N. Ü. Önal U. Merdan O. Akca B. Ener B. Özakın C. (2024). Synergistic antibacterial activity of ceftazidime-avibactam in combination with colistin, gentamicin, amikacin, and fosfomycin against carbapenem-resistant Klebsiella pneumoniae. Sci. Rep. 14 (1), 17567. 10.1038/s41598-024-67347-5 39080317 PMC11289488

[B24] Vincent J. L. Marshall J. Silva E. Anzueto A. Martin C. D. Moreno R. (2009). International study of the prevalence and outcomes of infection in intensive care units. JAMA 302, 2323e9. 10.1001/jama.2009.1754 19952319

[B25] Wang J. He J. T. Bai Y. Wang R. Cai Y. (2018). Synergistic activity of colistin/fosfomycin combination against carbapenemase-producing *Klebsiella pneumoniae* in an In Vitro pharmacokinetic/pharmacodynamic model. Biomed. Res. Int. 2018, 5720417. 10.1155/2018/5720417 29850537 PMC5937563

[B26] World Health Organization (2017). Global priority list of antibiotic-resistant bacteria to guide research, discovery, and development of new antibiotics. Available online at: https://www.who.int/medicines/publications/WHO-PPL-Short_Summary_25Feb-ET_NM_WHO.pdf?ua=1 (Accessed February 1, 2019).

[B27] Yanagida C. Ito K. Komiya I. Horie T. (2004). Protective effect of fosfomycin on gentamicin-induced lipid peroxidation of rat renal tissue. Chem. Biol. Interact. 148 (3), 139–147. 10.1016/j.cbi.2004.05.005 15276870

[B28] Yoshiyama Y. Yazaki T. Wong P. C. Beauchamp D. Kanke M. (2001). The effect of fosfomycin on glycopeptide antibiotic-induced nephrotoxicity in rats. J. Infect. Chemother. 7 (4), 243–246. 10.1007/s101560170020 11810591

